# Determination of common organophosphorus pesticides in the blood of children with acute leukaemia using a double-solvent system as a novel extractant for dispersive liquid–liquid microextraction

**DOI:** 10.1039/d0ra09303c

**Published:** 2020-12-18

**Authors:** Reza Akramipour, Mohammad Reza Golpayegani, Mostafa Sedighi, Farshad Fattahi, Nazir Fattahi

**Affiliations:** School of Medical, Kermanshah University of Medical Sciences Kermanshah Iran; Clinical Research Development Center, Mohammad Kermanshahi Hospital, Kermanshah University of Medical Sciences Kermanshah Iran; Research Center for Environmental Determinants of Health (RCEDH), Health Institute, Kermanshah University of Medical Sciences Kermanshah Iran n.fattahi@kums.ac.ir +988338263048 +989183364311

## Abstract

In this research, a new mode of dispersive liquid–liquid microextraction based on a double-solvent system (DLLME-DSS) was developed for the extraction and preconcentration of organophosphorus pesticides (OPPs) in the blood of children with acute leukaemia prior to determination by high-performance liquid chromatography-ultraviolet detection (HPLC-UV). In the present method, two long normal chain alcohols are mixed in a particular ratio, and then injected into the sample solution, which is magnetically stirred. In this case, the mixture of the two alcohols changes to a new aggregate extractant. This new double-solvent is used as an extractant, which has a higher extraction power than any of its components alone. Under the optimum conditions, the calibration graph was linear in the rage of 3–600 μg L^−1^ with detection limits of 1 to 2 μg L^−1^. Relative standard deviations (RSDs) including intra-day and inter-day of the method based on 7 replicate determinations of 100.0 μg L^−1^ for each analyte were in the range of 2.9–4.7% and 3.8–6.1%, respectively. The results proved that DLLME-DSS is a sensitive, very simple, inexpensive, environmentally friendly, rapid and efficient method for the preconcentration of trace amounts of OPPs in blood samples.

## Abstract 2

In this research, a new mode of dispersive liquid–liquid microextraction based on a double-solvent system (DLLME-DSS) was developed for the extraction of organophosphorus pesticides (OPPs) in the blood prior to determination by HPLC-UV.

## Graphical abstract

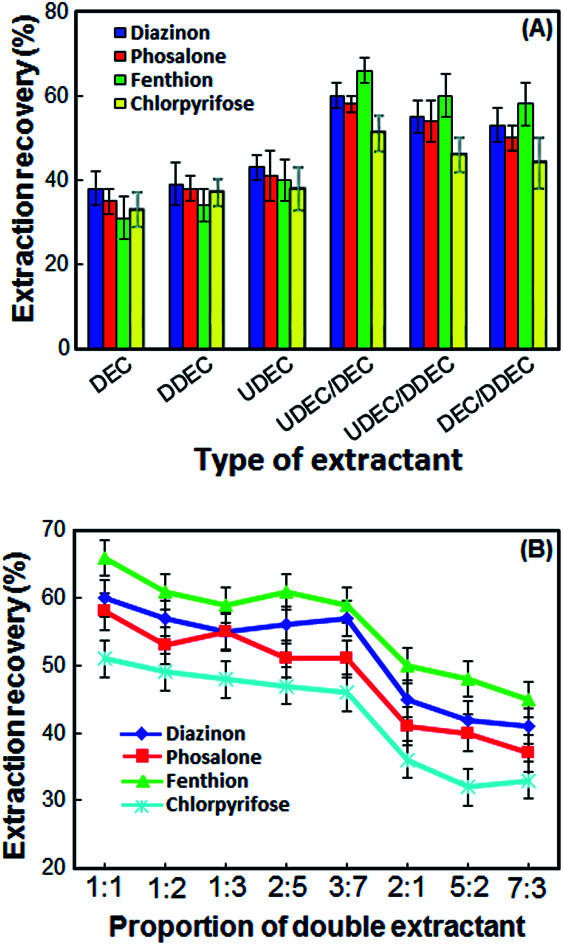

## 1 Introduction

Organopesticides are chemicals used to kill and control pests, diseases, weeds and microbes, and are most commonly used in agricultural products.^1^ Contact with pesticides can cause symptoms such as intoxication in the short term and complications such as neurological problems and cancers in the long term.^2^ Due to the widespread use of pesticides and the spread of toxic particles, issues such as the general health of people, especially children and infants, are raised even if they are not directly exposed to them. Studies show that children are more prone to being affected by toxin residues due to physiological deficiencies and rapid organ development.^3^ They also eat, drink and breathe more than adults based on their weight. Therefore, during rapid growth, especially in infancy and puberty, residual carcinogenic toxins and pesticides can have a greater effect on their cells, tissues, and other organs.^4^ In addition, pesticide residues are widely present in food products, and some pesticides can increase or decrease the effectiveness of medications.^5^ It may also increase the risk of infertility or stillbirth, brain and psychological problems, cancers and respiratory defects in the baby.^6^ Numerous previous studies suggest that exposure to pesticides is a risk factor for acute lymphocytic leukaemia in childhood.^7–10^ Due to contradictory studies on the relationship between pesticides and cancer incidence, this study will be conducted to investigate the level of organophosphate pesticides in children's blood and its relationship with acute lymphocytic cancer in Kermanshah province.

Analytical instrumentals such as high performance liquid chromatography (HPLC),^11–13^ gas chromatography (GC)^14–16^ and gas chromatography-mass spectrometry (LC-MS),^17–19^ have been used for the determination of OPPs in different matrices. GC-MS is usually employed for determination of OPPs because of high sensitivity, but due to the high cost, the use of this technique is limited. On the other hand, the HPLC-UV device is known to be simple, inexpensive, and found in most laboratories. Isolation and extraction of OPPs is an important stage for their determination in biological fluids. However, spite the use of a suitable analytical instrument, an extraction procedure is required before the OPPs analysis.

The sample preparation step is the most critical part of the analysis process, often associated with spending much time, high organic solvents consumption, and inaccuracy. To overcome these problems, miniature extraction techniques based on liquid–liquid extraction (LLE) have been developed today. Dispersive liquid–liquid microextraction (DLLME) is one of the best of these methods, that was first developed by Assadi *et al.*^20^ The advantages and disadvantages of this technique are mentioned in the articles.^21–24^ High consumption of disperser solvent (in milliliters) and selection of extraction solvent are problems of DLLME.^25,26^ To overcome these problems, innovations have been made on DLLME, such as DLLME based on solidification of floating organic droplet (SFO)^27–29^ and DLLME based on the deep eutectic solvent (DES).^30–33^ These innovations are aimed at further reducing the consumption of organic solvents and using organic extraction solvents lighter than water with less toxicity, cheaper, and more environmentally friendly.

In this study, a double-solvent system (DSS) was developed as an extractant without disperser solvent for DLLME. In this method, the first two long normal chain alcohols are mixed in a particular ratio, and then it is injected into the sample solution, which is on the magnetic stirrer. In this case, the mixture of the two alcohols is changed to new aggregate extractant and dispersed in tiny droplets with a very high contact surface in the sample solution. In this case, the extraction of analytes with different polarities is done through intermolecular hydrogen bonding or high-efficiency hydrophobic interaction. When the stirring stops, the extractant (DSS), which also contain analytes slowly accumulate on the surface of the sample solution and float in a droplet without the need for centrifugation. In the following, the mixture is put in the ice-bath for a 5 min to solidify the organic solvent, and the solid organic phase is transferred to a clean test tube. In this case, the collected organic phase melts easily at room temperature (RT) and the extractant is injected onto the HPLC-UV. The DLLME-DSS was evaluated to determine the OPPs in blood of children with acute leukaemia.

## 2 Experimental

### 2.1 Reagents and solutions

Diazinon, chlorpyrifos, fenthion and phosalone with a certified purity > 98% were purchased from polyscience (Niles, USA). The OPPs stock standard solution was prepared in methanol at the concentration level of 1000 mg L^−1^ and was stored at −20 °C. A fresh 2.0 mg L^−1^ of OPPs standard solution was prepared in methanol every week and stored at 4 °C. The ultra-pure water was purchased from Shahid Ghazi Pharmaceutical Co. (Tabriz, Iran). Methanol, acetonitrile, 1-undecanol, 1-dodecanol, 1-decanol, sodium dodecyl sulphate (SDS), Na_2_HPO_4_ and NaCl were obtained from Merck (Darmstadt, Germany).

### 2.2 Instrumentation

The analysis of OPPs were achieved on a HPLC Knauer equipped with a quaternary pump, online degasser, detector Smartline-UV-2500 variable wavelength programmable (Berlin, Germany) and a 20 μL injection loop injector (model 7725i, Rheodyne, Cotati, CA, USA). Separation was carried out with H5-ODS C18 column (15 cm × 4.6 mm, with 5 μm particle size) from Anachem (Luton, UK), preceded by a Security Guard Cartridge C_18_ (Anachem, Luton, UK). Separation of OPPs was performed with an elution gradient programmed linearly from an initial mobile phase composition of 50 : 50 (v/v) methanol : water to a final composition of 100% methanol at a flow rate of 1 mL min^−1^ and the analytes were detected at 254 nm. The Metrohm pH meter Model 692 (Herisau, Switzerland) was used for the pH values measurement.

### 2.3 Sampling and preparation of sample

Plasma samples were taken from patients with acute leukemia who were admitted and treated in the Dr Mohammad Kermanshahi Hospital from Kermanshah, Iran. In this way 3 girls and 3 boys (aged 3 to 11 years) were randomly chosen and 1.0 mL blood of each of them was taken and transferred to advanced research laboratory. For preparation and cleanup of samples, 400 μL of whole blood was placed in EDTA-contained glass test tube and one mL mixture of acetonitrile and ZnSO_4_ (15%, w/v) (2 : 3) was added to a test tube and vertex for 10 min. After holding the test tube at 4 °C for few minutes, it was centrifuged at 6000 rpm for 5 min. The obtained supernatant was transferred to another clean tube and was reached to a volume of 5 mL by distilled water to reduce the effects of matrices. The resulting solution was then subjected to the DLLME-DSS procedure.

### 2.4 Extraction procedure

A volume of 5.0 mL of a pretreated and diluted blood sample (spiked or not with OPPs) was placed in a 10 mL sample vial and fixed on a magnetic stirrer. Fifty microliter of double extractant (1-undecanol/1-decanol; 1 : 1 v/v) was injected into the diluted and pretreated blood sample, and the magnetic stirrer was turned on at 1200 rpm for 30 min. The extractant dispersed in tiny droplets with a very high contact surface in the sample solution without the need for a disperser solvent. In this case, the extraction of analytes is done through intermolecular hydrogen bonding or high-efficiency hydrophobic interaction. When the stirring stops, the extractant, which also contain analytes slowly accumulate on the surface of the sample solution and float in a droplet. The sample vial was thereafter put into an ice bath for five minutes; at this time, the floated extractant was solidified because of the low melting point. The solidified extractant was transferred into a conical vial where it was melted at room temperature. Finally, 30 μL of the solution was used for HPLC analysis.

## 3 Results and discussion

### 3.1 Double extractant type

**Fig. 1:**
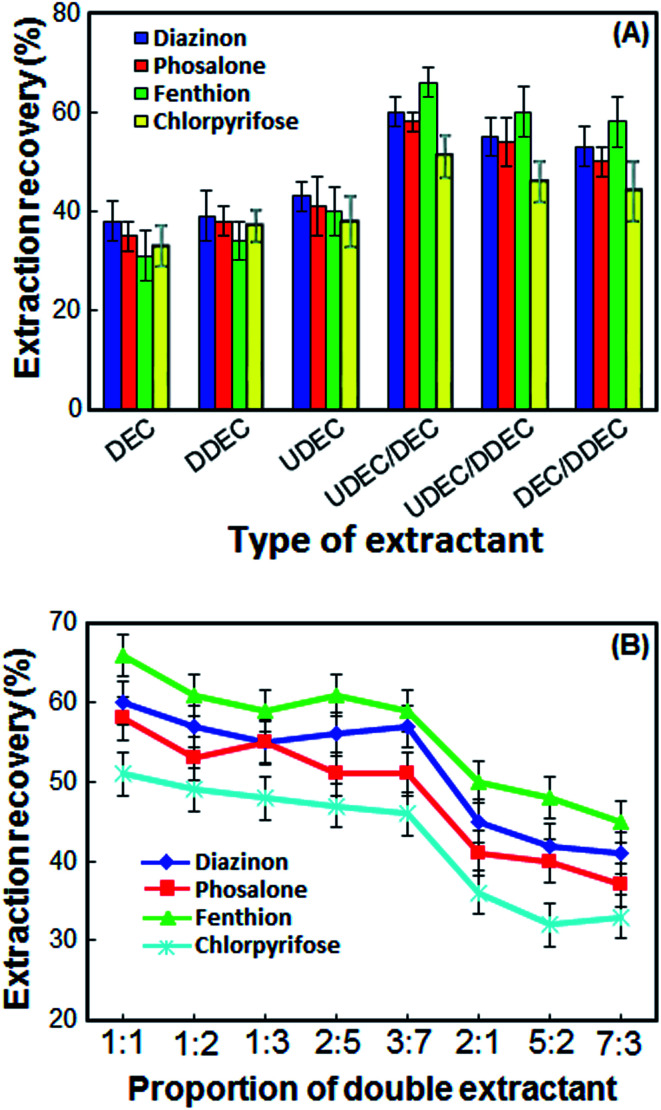
Effect of the different types of extractant (A) and proportion of double extractant (B) on the extraction recovery of the OPPs. Extraction conditions: volume of the sample solution, 5 mL; volume of the extraction solvent, 50 μL; stirring speed, 1200 rpm; extraction time, 30 min; room temperature.

Selection of double extractant is crucial in the DLLME-DSS. In this method for obtaining an appropriate double extractant, 1-undecanol, 1-decanol and 1-dodecanol solvents were selected. At first, each of the solvents was used as an extraction solvent, and then their double mixture with a specific ratio was used. The extraction recoveries (ER%) and standard deviations (SD) of OPPs using different extractants alone and double extractants are shown in Fig. 1A. According to the results in Fig. 1A, the ER% of OPPs with each solvent alone is not more than 40%, but when two of the solvents are mixed in a particular ratio, the ER% increases. Although the extraction recoveries of the OPPs by all double extraction systems are close to each other, in the 1-undecanol/1-decanol double extraction system, the extraction recoveries of OPPs are slightly better, and the standard deviation is low. Thus, 1-undecanol/1-decanol double system was chosen as the optimal double extraction system.

### 3.2 Proportion of double extractant

In the present work, the double extractants were obtained by using 1-undecanol and 1-decanol with different ratios (1 : 1, 1 : 2, 1 : 3, 2 : 5, 3 : 7, 2 : 1, 5 : 2 and 7 : 3) and some experiments were performed with these extractant. The results in Fig. 1B show that 1-undecanol and 1-decanol at a 2 : 1, 5 : 2 and 7 : 3 molar ratios could not form DSS. The mixture of 1-undecanol and 1-decanol in other ratios has a positive effect on the recovery of the OPPs. However, double extractant obtained from a mixture of 1-undecanol and 1-decanol in a 1 : 1 ratio, has higher ER% and lower SD. Therefore, the 1 : 1 ratio was chosen as the best ratio of 1-undecanol and 1-decanol.

### 3.3 Selection of double extractant volume

**Fig. 2:**
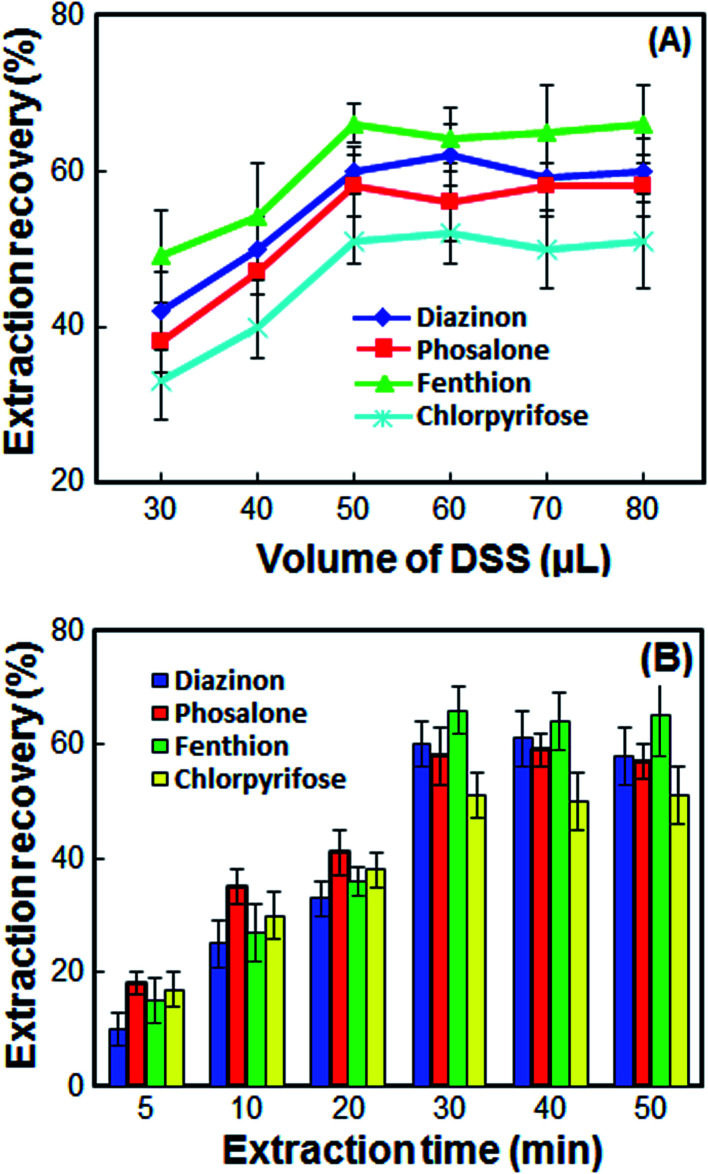
Effect of the volume of DSS (A) and extraction time (B) on the extraction recovery of the OPPs. Extraction conditions: as in Fig. 1; types of extractant, 1-undecanol/1-decanol; proportion of 1-undecanol/1-decanol, 1 : 1.

The double extractant volume plays a significant role in the extraction of OPPs. To study the effect of double extractant volume on the extraction recovery of OPPs, different double extractant volumes including 30, 40, 50, 60, 70 and 80 μL were tested to select the optimum volume of double extractant. According to the results in Fig. 2A, at volumes less than 50 μL, the contact surface is not high, and the extraction recovery is low. Also, not enough volume is obtained for injection into the HPLC, and the repeatability is significantly reduced. At volumes more than 50 μL, the extraction recoveries remain almost constant while the enrichment factor decreases sharply. Also, at high volume more than 50 μL, the double extractant cannot quickly accumulate on the surface of the sample solution. Thus, in order to have a high EF and good repeatability, 50 μL of double extractant was chosen as the optimum volume.

### 3.4 Effect of sample solution pH

In DLLME-DSS method, for investigating the effect of sample solution pH on the extraction recovery of OPPs, various experiments were performed by different pH of sample solution from 1 to 7 using HCl and acetate buffer. Other experimental conditions were kept constant. The results showed that the pH of sample solution has no significant effect on the extraction recovery of OPPs. So, the use of an acidic or buffer solution for adjust the pH, being the contamination sources, was not necessary.

### 3.5 Salt addition

For investigation of the salt addition on the performance of DLLME-DSS, various experiments were carried out by different amounts of NaCl (0–10%). The results indicated that with increasing NaCl from 0 to 5%, the extraction recovery of OPPs remain nearly constant, because there are two opposing factors that counteract each other. On the one hand the salting-out effect occurs which increases the ER% and on the other hand decreasing the solubility of the organic extraction phase in the sample solution increases the volume of the extraction phase and due to the dilution effect the enrichment factor decreases. At concentrations higher than 5%, the dilution effect prevails on salting-out effect and the ER% decreases. Therefore, the experiments were carried out in the absence of any salt.

### 3.6 Effect of extraction time

In this method, extraction time is defined as the time between injection of DSS and turn off the magnetic stirrer. The extraction time must be high enough to achieve an effective extraction of the analytes. On the other hand, it must be low enough not to waste time. So, the effect of extraction time on the ER% of OPPs was examined in the range of 5–50 min. When the extraction time increased from 5 to 30 min, the extraction of OPPs was increased due to the mass transfer of analyte from cellular material to DSS by diffusion and osmosis. However, the extraction recovery is kept constant upon further increase in extraction time (Fig. 2B). Therefore, the extraction time of 30 min was chosen as the optimum extraction time.

### 3.7 Effect of stirring speed

Stirring can improve the recovery of the OPPs in the DSS due to the mass transfer and dispersion of the DSS into the aqueous solution. For this purpose, different stirring speeds including 700, 800, 900, 1000, 1200, 1500 and 1800 rpm were investigated. As the stirring speed increases, the ER% of OPPs improves. However, if the stirring speed is too high, the solution will spatter and organic droplets will be destroyed. The stirring speed of 1200 rpm obtained the highest ER%. Thus, 1200 rpm was selected as the optimum stirring speed.

### 3.8 Quantitative analysis

The DLLME-DSS method was validated with respect to limit of detection (LOD), limit of quantification (LOQ), linear dynamic range (LDR), repeatability (intra-day), reproducibility (inter-day), extraction recovery (ER) and enrichment factor (EF). The characteristics of the calibration curve summarized in Table 1. Linear dynamic range was observed in the range of 3–600 μg L^−1^. Determination coefficients (*r*^2^) ranged from 0.986 to 0.998. Relative standard deviations (RSDs) including intra-day and inter-day of method based on 7 replicate determinations of 100.0 μg L^−1^ for each analyte were in the range of 2.9–4.7% and 3.8–6.1%, respectively. The LOD (S/N = 3) and LOQ (S/N = 10) were in the range of 1–2 μg L^−1^ and 3–6 μg L^−1^, respectively. The EF and ER% of the method were in the range of 73–94 and 51–66%, respectively, at the concentration level of 100 μg L^−1^ of OPPs and the sample volume of 5 mL.

**Table 1:** Quantitative result of DLLME-DSS and HPLC-UV of OPPs from blood sample

Analyte	ER%	EF	RSD% (intra-day, *n* = 7)	RSD% (inter-day, *n* = 7)	LDR (μg L^−1^)	*r* ^2^	LOD (μg L^−1^)	LOQ (μg L^−1^)
Diazinon	60	86	3.4	4.5	3–500	0.995	1	3
Phosalone	58	83	4.1	5.3	3–500	0.992	1	3
Fenthion	66	94	2.9	3.8	3–500	0.998	1	3
Chlorpyrifos	51	73	4.7	6.1	6–600	0.986	2	6

### 3.9 Analysis of blood samples

**Fig. 3:**
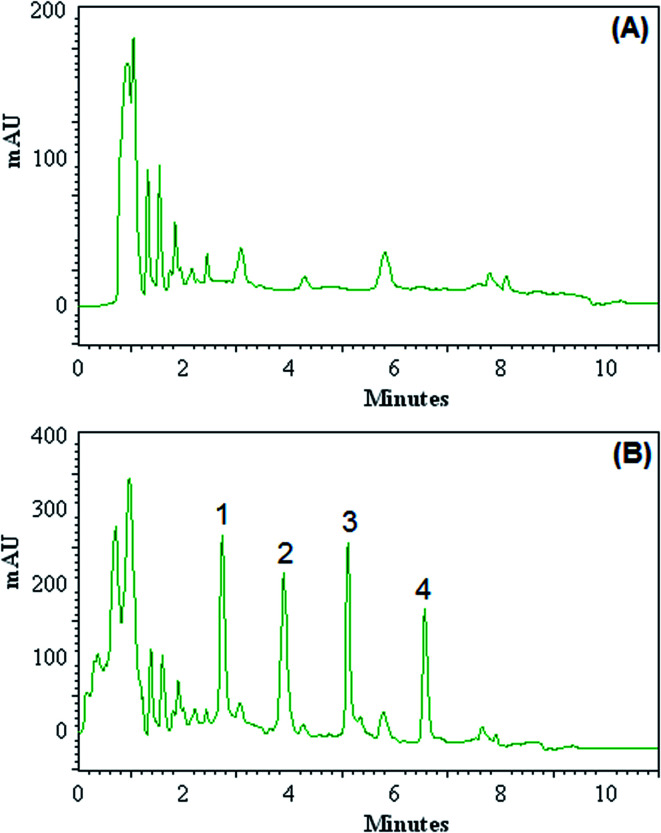
Chromatograms of blood sample taken from 5 year-old boy (A) and the corresponding spiked ones at concentration level of 100.0 μg L^−1^ for each of OPPs (B) obtained by using DLLME-DSS combined HPLC-UV. Peak identification: (1) diazinon, (2) phosalone, (3) fenthion, (4) chlorpyrifose.

The efficiency of the DLLME-DSS procedure with HPLC-UV instrument was validated with the monitoring of the OPPs in blood of patients with acute leukaemia. Blood samples were taken from patients with acute leukaemia who were admitted to and treated at Dr Mohamad Kermanshahi Hospital. The results showed that the analyzed samples were free of OPPs contamination. These samples were spiked with the standards of OPPs at different concentration levels to assess matrix effects. Fig. 3 shows the obtained chromatograms of blood sample taken from 5 year-old boy (A) and the corresponding spiked ones at concentration of 100.0 μg L^−1^ for each OPPs (B). Relative recovery of OPPs in spiked samples at different concentrations is shown in Table 2, ranging from 92 to 108%. These results demonstrate that the blood matrices, in our present context, have no significant effect on DLLME-DSS-HPLC-UV for determination of OPPs.

**Table 2:** Relative recoveries and standard deviations of OPPs from spiked blood samples^a^

Sample	Analyte	Added (μg L^−1^)	Found (mean ± SD^b^) (μg L^−1^)	Relative recovery (%)
Taken from a 9 year-old girl	Diazinon	10	9.6 ± 0.5	96
	Phosalone	10	10.3 ± 0.8	103
	Fenthion	10	9.2 ± 0.6	92
	Chlorpyrifos	10	10.1 ± 1.0	101
Taken from a 6 year-old girl	Diazinon	20	21.2 ± 1.3	106
	Phosalone	20	19.2 ± 1.6	96
	Fenthion	20	20.7 ± 1.8	103.5
	Chlorpyrifos	20	20.8 ± 2.0	104
Taken from a 3 year-old girl	Diazinon	30	31.0 ± 2.3	103
	Phosalone	30	29.6 ± 2.8	97
	Fenthion	30	28.2 ± 1.7	94
	Chlorpyrifos	30	32.5 ± 2.5	108
Taken from a 11 year-old boy	Diazinon	40	40.8 ± 3.5	102
	Phosalone	40	38.7 ± 2.6	97
	Fenthion	40	41.4 ± 3.2	103.5
	Chlorpyrifos	40	38.8 ± 3.6	97
Taken from a 7 year-old boy	Diazinon	50	53.2 ± 3.5	106
	Phosalone	50	52.2 ± 4.2	104
	Fenthion	50	49.5 ± 3.7	99
	Chlorpyrifos	50	47.4 ± 3.8	95
Taken from a 5 year-old boy	Diazinon	100	102.5 ± 5.2	102.5
	Phosalone	100	97.1 ± 6.3	97
	Fenthion	100	101.0 ± 5.8	101
	Chlorpyrifos	100	98.8 ± 4.1	99

a These data are based on the diluted volumes of blood samples and dilution effect was considered for calculation of them.

b Standard deviation.

### 3.10 Comparison of DLLME-DSS with other methods

The DLLME-DSS combined with HPLC-UV is compared with other procedures for determination of OPPs in different samples and the results are summarized in Table 3. According to Table 3, the detection limit of DLLME-DSS combined with HPLC-UV is lower than other techniques (except for GC-FPD) and consumption of toxic organic phase is greatly reduced. The RSD and linear range of the DLLME-DSS are superior to those reported before. Compared to other methods, the extraction time is relatively short except for the conventional DLLME. However, unlike the conventional DLLME method, in this method the disperser solvent is not required and no centrifuge is required for the separation of phases. All these results indicate that DLLME-DSS is a simple, inexpensive and reproducible technique that can be used for the extraction and preconcentration of OPPs in blood samples.

**Table 3:** Comparison of DLLME-DSS with other extraction methods for determination of OPPs in different samples

Methods	LOD^a^ (μg L^−1^)	LR^b^ (μg L^−1^)	RSD^c^ %	Extraction solvent volume	Extraction time (min)	Samples	Reference
LLE-LC-MS^d^	125–500	250–8000	1.5–8.4	200 μL	<10	Human serum	17
MSPE-GC-FPD^e^	0.21–2.28	1–100	1.8–8.7	400 μL	<15	Human hair and urine	14
HS-SPME-GC-PD^f^	2–55	20–20 000	0.9–9	Solvent free	<20	Biological samples	15
UASE-DLLME-GC-FPD^g^	0.1–0.5	0.5–1000	<10	5 mL + 60 μL	45	Tomato	1
UASE-DLLME-SFO-HPLC-UV^h^	1–4	5–800	≤9	5 mL + 150 μL	35	Fruit and vegetables	11
SPE-GC-FPD^i^	0.10–0.80	—	2.3–19.5	7 mL	∼15	Plasma and breastmilk	16
DLLME-DSS-HPLC-UV	1–2	3–600	2.9–4.7	50 μL	40	Blood of children	This work

a LOD, limit of detection.

b LR, linear range.

c RSD, relative standard deviation.

d Liquid–liquid extraction-liquid chromatography-mass spectrometry.

e Magnetic solid-phase extraction-gas chromatography-flame photometry detector.

f Headspace-solid phase microextraction-gas chromatography-nitrogen phosphorus detection.

g Ultrasonic assisted solvent extraction-dispersive liquid–liquid microextraction-gas chromatography-flame photometric detector.

h Ultrasonic assisted solvent extraction-dispersive liquid–liquid microextraction-solidification of floating organic drop-high performance liquid chromatography-ultraviolet detector.

i Solid-phase extraction-gas chromatography-flame photometry detector.

#### Live subject statement

The authors state that all experiments were performed in compliance with the relevant laws and institutional guidelines. The research ethics committee of Kermanshah University of Medical Sciences has approved the experiments about live subjects (Code of Ethics: IR.KUMS.REC.1398.1016). The authors also state that informed consent was obtained for any experimentation with human subjects and Kermanshah University of Medical Sciences is committed to the protection and safety of human subjects involved in research.

## 4 Conclusions

In this study for the first time, a double-solvent system as a novel extractant for dispersive liquid–liquid microextraction combined with HPLC-UV has been proposed for the determination of OPPs in blood of children with acute leukaemia. The advantages of this method include a simple operational procedure, inexpensive, environmental friendly, dispersive-solvent-free and low organic solvent consumption. We expected this method will be a breakthrough in separation science for the extraction of various organic compounds in blood samples. In this method, the first two long normal chain alcohols are mixed in a particular ratio, and then it is injected into the sample solution, which is on the magnetic stirrer. In this case, the mixture of the two alcohol changes to new extractant aggregate. This new DSS is used as an extractant, which has a higher extraction power than any of its components alone.

## Conflicts of interest

There are no conflicts to declare.

## Supplementary Material
